# Comparison of the incidence of periprocedural myocardial infarction between percutaneous coronary intervention with versus without rotational atherectomy using propensity score-matching

**DOI:** 10.1038/s41598-021-90042-8

**Published:** 2021-05-27

**Authors:** Yusuke Mizuno, Kenichi Sakakura, Hiroyuki Jinnouchi, Yousuke Taniguchi, Takunori Tsukui, Kei Yamamoto, Masaru Seguchi, Hiroshi Wada, Hideo Fujita

**Affiliations:** Division of Cardiovascular Medicine, Saitama Medical Center, Jichi Medical University, 1-847, Amanuma, Omiya-ku, Saitama, 330-8503 Japan

**Keywords:** Cardiology, Interventional cardiology

## Abstract

Complications such as slow flow are frequently observed in percutaneous coronary intervention (PCI) with rotational atherectomy (RA). However, it remains unclear whether the high incidence of slow flow results in the high incidence of periprocedural myocardial infarction (PMI), reflecting real myocardial damage. The aim of this study was to compare the incidence of PMI between PCI with versus without RA using propensity score-matching. We included 1350 elective PCI cases, which were divided into the RA group (n = 203) and the non-RA group (n = 1147). After propensity score matching, the matched RA group (n = 190) and the matched non-RA group (n = 190) were generated. The primary interest was to compare the incidence of PMI between the matched RA and non-RA groups. Before propensity score matching, the incidence of slow flow and PMI was greater in the RA group than in the non-RA group. After matching, the incidence of slow flow was still greater in the matched RA group than in the matched non-RA group (16.8% vs. 9.5%, *p* = 0.048). However, the incidence of PMI was similar between the matched RA and matched non-RA group (7.4% vs. 5.3%, *p* = 0.528, standardized difference: 0.086). In conclusion, although use of RA was associated with greater risk of slow flow, use of RA was not associated with PMI after a propensity score-matched analysis. The fact that RA did not increase the risk of myocardial damage in complex lesions would have an impact on revascularization strategy for severely calcified coronary lesions.

## Introduction

Rotational atherectomy (RA) is an essential procedure for the treatment of heavily calcified coronary lesions in percutaneous coronary intervention (PCI)^1,2^. However, the incidence of some complications, especially coronary perforation, is reported to be greater in PCI with RA than without^3–5^. Of those complications, slow flow is the most common complications following RA^6,7^, suggesting the incidence of slow flow would be higher in PCI with RA than without. However, it remains unclear whether the higher incidence of slow flow results in the higher incidence of periprocedural myocardial infarction (PMI), which reflects real myocardial damage and is closely associated with future cardiovascular events including cardiac death^8–12^. It is of importance to confirm whether RA increases the incidence of PMI, because interventional cardiologists would hesitate to select RA among several specific procedures including orbital atherectomy and intravascular lithotripsy for severely calcified lesions if RA increases the incidence of PMI considerably.

Although a few studies suggested the greater incidence of PMI in PCI with RA than without, the lesion characteristics such as the degree of calcification were more complex in PCI with RA than without^3,13,14^, which would make it difficult to compare the incidence of PMI between PCI with and without RA. Even randomized studies may not answer clearly whether the incidence of PMI was greater in PCI with RA, because the prevalence of crossover from PCI without RA to PCI with RA was quite high (12.5–16.0%) in recent randomized trials comparing PCI with versus without RA^15,16^, which is much higher rates of crossover compared to other randomized studies in the field of PCI^17,18^. Considering these specific background regarding PCI with RA, we hypothesized that a propensity score-matched analysis would work for adjusting clinical and lesion characteristics between PCI with versus without RA. The aim of this study was to compare the incidence of PMI between PCI with versus without RA using a propensity score-matched analysis.

## Methods

### Study design

This was a retrospective, single-center study. We reviewed consecutive PCI cases in the Saitama Medical Center, Jichi Medical University from January 2018 to March 2020. The inclusion criterion was (1) elective PCI cases that were performed during the study period. Elective PCI cases included the culprit lesions of acute myocardial infarction as long as PCI was planned as elective PCI. The exclusion criteria were (1) cases which did not have either creatine kinase (CK) or CK-myocardial band (MB) values at the next day of PCI, (2) cases in which ≥ 2 vessels were treated simultaneously, and (3) saphenous vein graft lesions. We divided the study cases into the lesions that were treated with RA (RA group) and the lesions that were treated without RA (non-RA group). Propensity score matching was performed to match the background characteristics between the 2 groups. The details of the propensity score-matched analysis are described in the statistical analysis section. Clinical characteristics and outcomes were compared between the 2 groups both before and after propensity score-matching. The primary interest in the present study was the incidence of PMI after a propensity score-matching. The secondary interests included the incidence of slow flow and other periprocedural complications. This study was approved by the Institutional Review Board of Saitama Medical Center, Jichi Medical University (S20-152), and written informed consent was waived by the institutional review board of Saitama Medical Center, Jichi Medical University, because of the retrospective study design. All methods were performed in accordance with the relevant guidelines and regulations.

### Definition

The definition of overweight, hypertension, dyslipidemia, diabetes mellitus, and chronic renal failure was described in elsewhere^19–22^. We also calculated the estimated glomerular filtration rate (eGFR) from the serum creatinine level, age, weight, and gender using the following formula: eGFR = 194 × Cr-1.094 × age-0.287 (male), eGFR = 194 × Cr-1.094 × age-0.287 × 0.739 (female)^20^. In patients whose baseline CK levels were normal, PMI was defined as an elevation of CK levels ≥ 2 times of the upper limit of normal (ULN) with an elevation of CK-MB levels above the ULN at the next day of PCI^23–26^. If baseline CK levels were already elevated, PMI was defined as further increase of CK levels at the next day of PCI than CK level at baseline^26^. We also checked PMI defined by the Society for Cardiovascular Angiography and Interventions (SCAI), and PMI defined by the EXCEL (Evaluation of XIENCE vs. Coronary Artery Bypass Surgery for Effectiveness of Left Main Revascularization)^8,27^.

### PCI procedures

The choice of PCI devices such as guide wire, balloon, rotational atherectomy and stent was left at the discretion of interventional cardiologists at our cardiology center. IVUS or OCT were routinely used for almost all lesions. In bifurcation lesions, we usually insert a conventional guidewire to a side branch before stenting to the main vessel, and occasionally perform jailed balloon technique/jailed corsair technique. We conducted a single stent technique, and seldom selected two-stent technique, especially in elective PCI. Successful PCI was defined as angiographical residual diameter stenosis < 50% with decrease in minimum stenosis with TIMI flow grade ≥ 2^28,29^.

Rotational atherectomy was performed to heavily calcified lesions, diffuse lesions expected to be difficult to stent, and ostial lesions^22^. We used the nicorandil based drug cocktail (nicorandil 12 mg, isosorbide dinitrate 2.5 mg, heparin 10,000 units, and normal saline 500 mL) for all RA cases. The lesion was crossed with a 0.014-inch conventional guidewire, which was exchanged with a 0.009-inch RotaWire floppy or RotaWire extra support guidewire (Boston Scientific, Natick, MA, USA) using a microcatheter. The RA burr was subsequently advanced over the wire to a position proximal to the lesion. The rotational speed was set at the conventional range (140,000–190,000 rpm) with the burr proximal to the lesion^30^. The burr was activated and moved forward with a slow pecking motion. Each run time was < 30 s, and care was taken to avoid a decrease in rotational speed > 5000 rpm. The presence of coronary flow was confirmed by injecting sufficient contrast medium immediately after the burr was pulled out.

Our university hospital had many operators including residents with different background. However, each PCI was strictly supervised by staff operator, and all PCI with RA were supervised by a senior staff operator (K. Sakakura). Our techniques regarding RA was almost consistent to those that were recommended by the clinical expert consensus document on RA from the Japanese association of cardiovascular intervention and therapeutics (CVIT)^2^. Staff operators did not hesitate to take over procedures, when residents felt any difficulties in procedures. Activated coagulation time was maintained over 250 s during procedures.

### Angiographical analysis

Quantitative coronary angiography parameters were measured using a cardiovascular angiography analysis system (QAngio XA 7.3, MEDIS Imaging Systems, Leiden, Netherlands). The lesion length and reference diameter were measured. Bifurcation lesions were divided into seven categories according to the Medina classification system^31^. In this system, we recorded any narrowing ≥ 50% in angiography in each of the three arterial segments of the bifurcation in the following order: proximal main vessel, distal main branch, and side branch: 1 is used to indicate the presence of a significant stenosis and 0 was used to indicate the absence of stenosis^31^. Calcification was identified as readily apparent radiopacities within the vascular wall at the site of the stenosis, and was classified as none/mild, moderate (radiopacities noted only during the cardiac cycle before contrast injection), and severe (radiopacities noted without cardiac motion before contrast injection generally compromising both sides of the arterial lumen)^32^. Slow flow was defined as slow or absent distal runoff (TIMI flow grade ≤ 2) just after RA, balloon dilation or deployment of stent^33^.

### Statistical analysis

Data are presented as a percentage for categorical variables, a mean ± standard deviation (SD) for normally distributed continuous variables, and median (quartile 1–quartile 3) for nonparametric variables. The Wilk-Shapiro test was performed to determine if the continuous variables were normally distributed. Normally distributed continuous variables were compared between the 2 groups using a Student’s t test. Otherwise, continuous variables were compared using a Mann–Whitney U test. Categorical variables were compared using a Fisher’s exact test. One-to-one propensity score matching was used to match the clinical background between the 2 groups. RA was set as a dependent variable, whereas parameters that were clinically relevant for complications in RA, such as age, male sex, overweight, hypertension, dyslipidemia, diabetes mellitus, estimated GFR, PCI indication for acute myocardial infarction, lesions at left main trunk and/or left anterior descending artery, chronic total occlusion, reference diameter, lesion length, lesion angle, calcification and bifurcation were set as independent variables^5^. For matching, the match tolerance was initially set as a width of 0.25 multiplied by the SD of the propensity score distribution^34,35^. However, the generated sample size was only 358 lesions (179 lesions in each group). In order to assess an appropriate sample size, we additionally performed the power analysis using the estimated incidences of PMI in PCI with RA as 9.7% and PCI without RA as 2.8%^14^. The required sample size was 384 lesions (192 lesions in each group) assuming as statistical power (1-β) of 80% and a sensitivity (α) of 5%. Therefore, we performed another matching using the more liberal match tolerance that was set as a width of 0.30 multiplied by the SD of the propensity score distribution, and the generated sample size was 380 lesions (190 lesions in each group). We adopted these 380 lesions (190 lesions in each group) as main analysis, and used 358 lesions (179 lesions in each group) as supplemental analysis. Balance between matched RA group and matched non-RA group after propensity score matching was assessed by using standardized difference of each covariate where a standardized difference < 0.1 was considered a negligible difference in the mean or prevalence of a covariate between RA group and non-RA group^36,37^.

Multivariate logistic regression analysis was performed to investigate associations between clinical variables including RA and PMI after propensity score matching analysis. In this model, PMI was used as the dependent variable. Variables that had a significant association (*p* < 0.05) between the matched 2 groups and use of RA were used as independent variables. The odds ratio (OR) and the 95% confidence interval (CI) were calculated. A *p* value < 0.05 was considered statistically significant. We analyzed all data by IBM SPSS statistics version 24 (Chicago, IL, USA).

## Results

**Figure 1:**
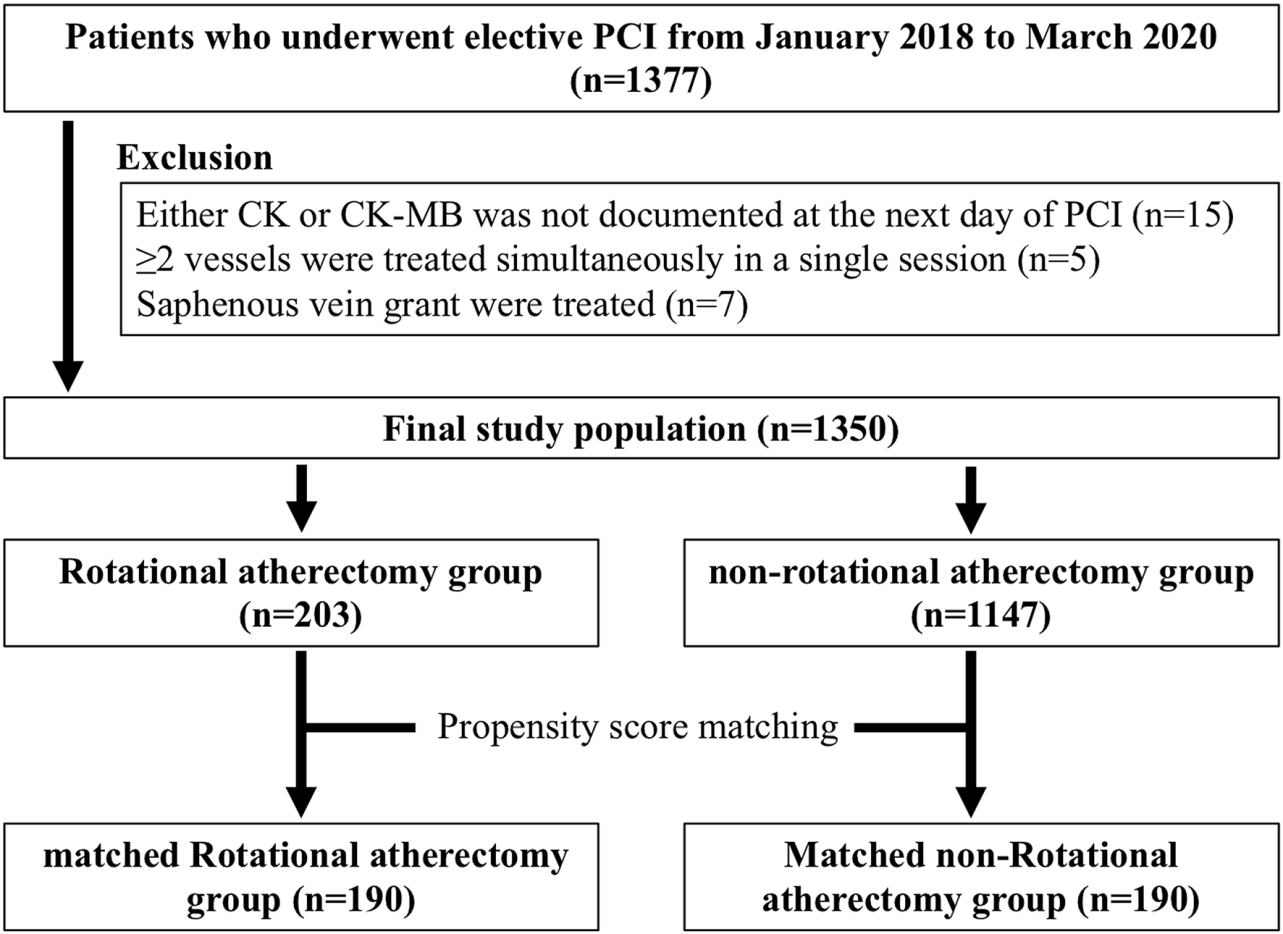
The study flow-chart. PCI = percutaneous coronary intervention; CK = creatinine kinase; CK-MB = creatinine kinase-myocardial band; RA = rotational atherectomy.

Overall, 1377 elective PCI cases were performed in our hospital from January 2018 to March 2020, and 27 PCI cases were excluded. Our final study population was 1350 PCI cases, which were divided into in the RA group (n = 203) and the non-RA group (n = 1147). After a propensity score-matched analysis, study population was divided into the matched RA group (n = 190) and matched non-RA group (n = 190). The study flowchart is shown as the Fig. 1.

Patient, lesion and procedural characteristics between the 2 groups before and after propensity score matching are summarized in Table 1. Although several patient’s characteristics were significantly different between the RA and non-RA groups before matching, all patient’s characteristics except diabetes mellitus were similar between the matched RA and matched non-RA groups.

**Table 1:** Comparison of patients, lesions, and procedural characteristics between the RA and the non-RA groups before and after propensity score matching using the match tolerance set as a width of 0.30 multiplied by the SD of the propensity score distribution.

	Before propensity score matching				After propensity score matching			
	RA group (n = 203)	Non-RA group (n = 1147)	*p* value	Standardized difference	Matched RA group (n = 190)	Matched non-RA group (n = 190)	*p* value	Standardized difference
**Patient characteristics**								
Age (years)	74 (62.0–80.0)	72 (64.0–77.0)	0.001	0.331	74.0 (69.0–84.0)	74.0 (69.0–84.0)	0.952	0.010
Men—n, (%)	145 (71.4)	932 (81.3)	0.002	0.235	138 (72.6)	143 (75.3)	0.640	0.062
Overweight (BMI ≥ 25 kg/m^2^)—n, (%)	57 (28.1)	414 (36.1)	0.031	0.172	50 (26.3)	50 (26.3)	1.000	0
Hypertension—n, (%)	186 (91.6)	1024 (89.3)	0.382	0.078	173 (91.1)	172 (90.5)	1.000	0.021
Diabetes mellitus—n, (%)	112 (55.2)	538 (46.9)	0.030	0.167	103 (54.2)	148 (77.9)	< 0.001	0.517
Insulin user—n, (%)	36 (32.1) (n = 112)	131 (24.3) (n = 538)	0.096	0.174	32 (31.1) (n = 103)	45 (30.4) (n = 148)	1.000	0.015
Dyslipidemia—n, (%)	178 (87.7)	963 (84.0)	0.206	0.106	165 (86.8)	166 (87.4)	1.000	0.018
Current smoker—n, (%)	20 (10.4) (n = 192)	200 (18.0) (n = 1111)	0.009	0.219	20 (11.1) (n = 180)	22 (12.0) (n = 184)	0.870	0.028
Chronic renal failure (creatinine > 2 mg/dl)—n, (%)	55 (27.1)	129 (11.2)	< 0.001	0.413	46 (24.2)	43 (22.6)	0.809	0.038
Estimated GFR (mL/min/1.73 m^2^)	53.5 (11.2–70.0)	62.9 (47.6–77.8)	< 0.001	0.433	53.9 (22.43–71.0)	53.1 (29.7–65.8)	0.770	0.021
Chronic renal failure on hemodialysis—n, (%)	53 (26.1)	108 (9.4)	< 0.001	0.448	45 (23.7)	34 (17.9)	0.206	0.143
Statin treatment—n, (%)	168 (82.8)	871 (75.9)	0.037	0.171	156 (82.9)	159 (83.7)	0.785	0.043
**Lesion characteristics**								
Reason for PCI			0.172	0.108			0.958	0.029
PCI to the culprit of ST elevation myocardial infarction—n, (%)	2 (1.0)	5(0.4)			2 (1.1)	2 (1.1)		
PCI to the culprit of non-ST elevation myocardial infarction—n, (%)	32 (15.8)	141 (12.3)			29 (15.3)	31 (16.3)		
PCI to the non-AMI lesions—n, (%)	169 (83.3)	1001 (87.3)			159 (83.7)	157 (82.6)		
Target lesion			< 0.001	0.948			0.103	0.137
Left main–left anterior descending artery—n, (%)	131 (64.5)	502 (43.8)			120 (63.2)	105 (55.3)		
Left circumflex artery—n, (%)	18 (8.9)	253 (22.1)			18 (9.5)	31 (16.3)		
Right coronary artery—n, (%)	54 (26.6)	392 (34.2)			52 (27.4)	54 (28.4)		
PCI to in-stent restenosis—n, (%)	15 (7.4)	125 (10.9)	0.168	0.122	14 (7.4)	22 (11.6)	0.220	0.144
PCI to chronic total occlusion—n, (%)	4 (2.0)	120 (10.5)	< 0.001	0.357	4 (2.1)	5 (2.6)	1.000	0.033
Reference diameter (mm)	2.39 (2.05–2.71)	2.37 (2.00–2.83)	0.853	0.012	2.38 (2.05–2.71)	2.35 (2.04–2.69)	0.714	0.042
Lesion length (mm)	18.78 (11.52–35.02)	14.84 (10.02–24.62)	< 0.001	0.301	17.79 (10.70–31.16)	18.06 (11.21–26.99)	0.513	0.111
Lesion angle			< 0.001	0.359			0.147	0.228
Mild (< 45°)	85 (41.9)	665 (58.0)			83 (43.7)	88 (46.3)		
Moderate (45°–90°)	83 (40.9)	375 (32.7)			75 (39.5)	83 (43.7)		
Severe (> 90°)	35 (17.2)	107 (9.3)			32 (16.8)	19 (10.0)		
Calcification			< 0.001	1.024			0.751	0.011
None/mild, n (%)	4 (2.0)	864 (75.3)			4 (2.1)	6 (3.2)		
Moderate/severe, n (%)	199 (98.0)	285 (24.8)			186 (97.9)	184 (96.8)		
Bifurcation lesion (n = 585)								
Medina classification			0.097	0.578			0.544	1.248
1,0,0	11 (8.3) (n = 132)	48 (10.6) (n = 453)			10 (8.4) (n = 119)	9 (8.7) (n = 103)		
1,0,1	7 (5.3) (n = 132)	19 (4.2) (n = 453)			7 (5.9) (n = 119)	2 (1.9) (n = 103)		
1,1,0	41 (31.1) (n = 132)	124 (27.4) (n = 453)			37 (31.1) (n = 119)	36 (35.0) (n = 103)		
1,1,1	18 (13.6) (n = 132)	67 (14.8) (n = 453)			17 (14.3) (n = 119)	14 (13.6) (n = 103)		
0,1,0	30 (22.7) (n = 132)	107 (23.6) (n = 453)			27 (22.7) (n = 119)	21 (20.4) (n = 103)		
0,1,1	23 (17.4) (n = 132)	54 (11.9) (n = 453)			19 (16.0) (n = 119)	15 (14.6) (n = 103)		
0,0,1	2 (1.5) (n = 132)	34 (7.5) (n = 453)			2 (1.7) (n = 119)	6 (5.8) (n = 103)		
**Procedural characteristics**								
Successful PCI	203 (100)	1134 (98.9)	0.237	0.149	190 (100)	190 (100)	–	–
Guiding catheter size			< 0.001	0.465			< 0.001	0.688
≤ 6Fr—n, (%)	4 (2.0)	638 (55.6)			4 (2.1)	76 (40.0)		
7Fr—n, (%)	180 (88.7)	427 (37.2)			167 (87.9)	110 (57.9)		
8Fr—n, (%)	19 (9.4)	82 (7.1)			19 (10.0)	4 (2.1)		
Intra-aortic balloon pump support—n, (%)	1 (0.5)	10 (0.9)	1.000	0.048	1 (0.5)	4 (2.1)	0.372	0.142
Use of orbital atherectomy—n, (%)	0 (0)	21 (1.8)	0.060	0.191	0 (0)	14 (7.4)	< 0.001	0.400
Use of scoring balloon or cutting balloon—n, (%)	73 (36.0)	301 (26.2)	0.004	0.213	71 (37.4)	86 (45.3)	0.145	0.161
Side branch protection			< 0.001	0.259			0.137	0
Jailed wire—n, (%)	81 (39.9)	262 (22.8)			74 (38.9)	54 (28.4)		
Jailed corsair—n, (%)	7 (3.4)	32 (2.8)			6 (3.2)	7 (3.7)		
Jailed balloon—n, (%)	2 (1.0)	8 (0.7)			1 (0.5)	1 (0.5)		
Total stent and DCB length	35.5 (20.0–48.5) (n = 202)	24.0 (18.0–38.0) (n = 1117)	< 0.001	0.386	32.0 (20.0–46.5) (n = 189)	30.0 (20.0–38.0) (n = 185)	0.094	0.152
Kissing balloon technique—n, (%)	0 (0)	12 (1.0)	0.232	0.142	0 (0)	2 (1.1)	0.499	0.149
Final PCI procedure			0.082	0.632			0.220	0.424
DES—n, (%)	170 (83.7)	936 (81.6)			158 (83.2)	153 (80.5)		
DCB—n, (%)	22 (10.8)	155 (13.5)			22 (11.6)	25 (13.2)		
DES + DCB—n, (%)	8 (3.9)	18 (1.6)			7 (3.7)	6 (3.2)		
BMS—n, (%)	2 (1.0)	7 (0.6)			2 (1.1)	0 (0)		
POBA—n, (%)	1 (0.5)	20 (1.7)			1 (0.5)	6 (3.2)		
Other—n, (%)	0 (0)	11 (1.0)			0 (0)	0 (0)		

Data are presented as a percentage for categorical variables or a median (quartile 1–quartile 3) for nonparametric variables. A Mann–Whitney U test was used for nonparametric continuous variables. A Fisher’s exact test was used for categorical variables.

RA, rotational atherectomy; BMI, body mass index; GFR, glomerular filtration rate; PCI, percutaneous coronary intervention; AMI, acute myocardial infarction; DCB, drug-coated balloon; TIMI, Thrombolysis in Myocardial Infarction; DES, drug-eluting stent; BMS, bare-metal stent; POBA, plain old balloon angioplasty.

Lesion characteristics were also similar between the matched RA and matched non-RA groups after matching. In procedural characteristics, the size of guiding catheter was larger in the matched RA group than in the matched non-RA group (*p* < 0.001). Orbital atherectomy was more frequently used in the matched non-RA group than in the matched RA group (*p* < 0.001). Furthermore, patient, lesion and procedural characteristics between the 2 groups after propensity score matching using the match tolerance that was set as a width of 0.25 multiplied by the SD of the propensity score distribution are summarized in Supplemental Table 1.

Complications between the 2 groups is shown in Table 2. The incidence of slow flow was significantly greater in the matched RA group (16.8%) than in the matched non-RA group (9.5%) (*p* = 0.048), whereas the incidence of PMI was similar between the 2 matched groups (7.4% vs. 5.3%, *p* = 0.528). Standardized difference of PMI in the 2 matched groups was 0.086. Furthermore, complications between the 2 groups after propensity score matching using the match tolerance that was set as a width of 0.25 multiplied by the SD of the propensity score distribution are shown in Supplemental Table 2.

**Table 2:** Comparison of complications between the RA and non-RA groups before and after propensity score matching using the match tolerance set as a width of 0.30 multiplied by the SD of the propensity score distribution.

	Before propensity score matching				After propensity score matching			
	RA group (n = 203)	Non-RA group (n = 1147)	*p* value	Standardized difference	Matched RA group (n = 190)	Matched non-RA group (n = 190)	*p* value	Standardized difference
Periprocedural myocardial infarction—n, (%)	16 (7.9)	31 (2.7)	0.001	0.234	14 (7.4)	10 (5.3)	0.528	0.086
Periprocedural myocardial infarction (SCAI definition)—n, (%)	0 (0)	2 (0.2)	1.000	0.707	0 (0)	1 (0.5)	1.000	0.100
Periprocedural myocardial infarction (EXCEL definition)—n, (%)	1 (0.5)	3 (0.3)	0.479	0.413	0 (0)	2 (1.1)	0.499	0.149
Creatine kinase (U/L) at the next day of PCI	98 (62.0–156.0)	80.0 (57.0–120.0)	< 0.001	0.194	98.0 (62.0–156.0)	86.0 (55.0–142.0)	0.149	0.070
Slow flow—n, (%)	34 (16.7)	101 (8.8)	0.001	0.239	32 (16.8)	18 (9.5)	0.048	0.217
Final TIMI grade of main vessel			0.242	1.029			0.123	1.414
TIMI3—n, (%)	202 (99.5)	1124 (98.0)			190 (100.0)	186 (97.9)		
≤ TIMI2 (%)—n, (%)	1 (0.5)	23 (2.0)			0 (0)	4 (2.1)		
Final TIMI grade of side branch (n = 219)			0.303	0.300			0.657	0.223
TIMI3—n, (%)	117 (88.6) (n = 132)	415 (91.6) (n = 453)			106 (89.1) (n = 119)	94 (91.3) (n = 103)		
≤ TIMI2 (%)—n, (%)	15 (11.4) (n = 132)	38 (8.4) (n = 453)			13 (10.9) (n = 119)	9 (8.7) (n = 103)		
Coronary perforation (Ellis type 3)—n, (%)	0 (0)	3 (0.3)	1.000	0.926	0 (0)	0 (0)	-	-
Device stuck—n, %	0 (0)	0 (0)	-	-	0 (0)	0 (0)	-	-

Data are presented as a percentage for categorical variables or a median (quartile 1- quartile 3) for nonparametric variables. A Mann–Whitney U test was used for nonparametric continuous variables. A Fisher’s exact test was used for categorical variables.

PCI, percutaneous coronary intervention, TIMI, Thrombolysis in Myocardial Infarction.

We performed a multivariate logistic regression analysis to confirm the relationship between use of RA and PMI in the matched study population. Other than RA, we included diabetes mellitus and guide catheter size, which were significantly different between the 2 matched groups, as independent variables. Although the use of orbital atherectomy was significantly different between the matched group, there was no periprocedural myocardial infarction in lesions required orbital atherectomy, which makes impossible to calculate a odds ratio. Therefore, orbital atherectomy was not included in the variable. Table 3 shows use of RA (OR 1.229; 95% CI 0.479 − 3.153; *p* = 0.667) was not significantly associated with PMI after controlling confounding factors (diabetes mellitus and guide catheter size).

**Table 3:** Multivariate logistic regression model to find the factors associated with periprocedural myocardial infarction in the matched RA and non-RA groups.

Independent variables	Odds ratio	95% confidence interval	*p* value
**Dependent variable: periprocedural myocardial infarction**			
Rotational atherectomy	1.229	0.479–3.153	0.667
Diabetes mellitus	1.128	0.457–2.783	0.794
Guide catheter size ≥ 7Fr (versus 6Fr or 5Fr)	1.735	0.448–6.718	0.425

In the multivariate logistic regression model, the selection of independent variables was derived from the results of matched groups; rotational atherectomy, diabetes mellitus and Guide catheter size.” Although the use of orbital atherectomy was significantly different between the matched group, there was no periprocedural myocardial infarction in lesions required orbital atherectomy, which makes impossible to calculate a odds ratio. Therefore, orbital atherectomy was not included in the variable.

## Discussion

We included 1350 elective PCI cases, which were divided into the RA group (n = 203) and the non-RA group (n = 1147). After propensity score matching, the matched RA group (n = 190) and the matched non-RA group (n = 190) were generated. The main findings of our study were as follows: (1) although the incidence of slow flow was greater in the matched RA group than in the matched non-RA group, the incidence of PMI was similar between the matched RA and matched non-RA group, suggesting RA would not increase the risk of PMI; and (2) other complications such as side branch occlusion, vessel perforation and device stuck were not different between the matched 2 groups.

We should discuss the discrepancy that the incidence of PMI was greater in the RA than in the non-RA group, whereas the incidence of PMI was similar between the matched RA and matched non-RA groups. Our group recently reported the determinants of PMI were complex lesion features such as diffuse long lesion, larger lesion angle or true bifurcation lesions^14^. The results before matching would suggest that the target lesions had more complex features such as calcification in PCI with RA than in PCI without RA. In fact, lesion length was significantly longer, lesion angle was significantly severer, and calcification was significantly severer in the RA group than in the non-RA group. Earlier retrospective studies comparing the incidence of complications between PCI with versus without RA could not adjust clinical background between PCI with and without RA^3,13^. Our propensity-score matching adjusted clinical and lesion characteristics well, and allowed us to compare the real additional risk of RA for complex lesions. Our results suggest that use of RA itself would not increase the risk of complications during PCI to complex lesions.

Of complications, only slow flow was more frequently observed in the matched RA group than in the matched non-RA group. There are several explanations why the incidence of slow flow was greater in the matched RA group. First, our group has been very careful about picking up slow flow during PCI with RA since November 2014, when our group started a randomized study regarding slow flow during RA(trial registration: UMIN 000015702). Indeed, our group has published several literatures regarding slow flow during RA^30,38,39^. Thus, we picked up mild slow flow (e.g. transient TIMI-2 flow only just after RA) as well as severe slow flow. The incidence of slow flow during RA varies widely from 2.7 to over 20%, depending on the definition, timing of judgement, and the length of target lesion^40–42^. Our incidence of slow flow in PCI with RA was relatively high as compared to literatures. Second, although we reviewed all cine-angiogram and catheter reports, we might not pick up slow flow in PCI without RA as we picked up slow flow in PCI with RA. Therefore, we might miss transient slow flow and underestimate the incidence of slow flow in PCI without RA.

Clinical implications of the present study should be noted. First, the greater incidence of PMI after RA was not attributed to use of RA itself, but to lesion complexity. We do not need to hesitate to use RA for complex lesions in fear of PMI or myocardial damage. The incidence of slow flow was greater in PCI with RA than in PCI without even after propensity score matching. RA operators should recognize the greater risk of slow flow during RA. However, if transient slow flow just after RA was treated adequately, most slow flow would not result in myocardial damage expressed as PMI. RA operators should be familiar with the prevention and bailout for slow flow to avoid myocardial damage that affects patient’s clinical outcomes^2^. Furthermore, the prevalence of chronic kidney disease was relatively high in our study population. Since earlier studies reported the high percentage of advanced atherosclerotic plaques such as large lipid volume or dense calcification in patients with chronic kidney disease^43–45^, those patients would have a greater risk of PMI. Thus, our study population was the high risk population for PMI. It is noteworthy that the incidence of PMI was comparable between the RA and non-RA groups in these high risk population.

### Study limitations

First, this study was as single-center retrospective observational study, there is a risk of patient selection bias and group selection bias. Furthermore, some important variables such as the duration of diabetes mellitus were not available because of the retrospective nature of this study. The frequency of RA usage was greater in our catheter laboratory than Japanese national PCI registry^5^, which could be an institutional bias. Furthermore, the incident of coronary perforation or device entrapment was 0% in the RA group, which was low as compared to literatures^3,15,46^. We might be familiar with various techniques such as halfway RA to prevent severe complications^47^. Second, our study population was not a patient-level database, but a PCI-level database. Therefore, we could not exclude the effect of the clustered nature of one or more individual measurements from one patient. Third, we did not adopt the definition of PMI by the universal definition [cardiac troponin > 5 times upper limit of normal and PCI-related clinical or angiographic complications], but adopted the definition of PMI by the rise of CK/CK-MB, because the definition of PMI by the universal definition might be influenced by the subjective judgment^11^. Fourth, we used lesion characteristics such as PCI indication for acute myocardial infarction, lesions at left main trunk and/or left anterior descending artery, chronic total occlusion, reference diameter, lesion length, lesion angle, calcification and bifurcation, which were drawn from angiographical findings. The matching of lesion characteristics might be insufficient by such angiographical findings, and might be improved by IVUS findings. However, it was difficult to use IVUS findings for matching, because pre-procedural IVUS could not cross the calcified lesion before RA in approximately half of cases. Finally, there was a possibility of a Type II error (ß error) in the comparison of complications between the 2 matched groups. However, the required sample size would be 384 lesions (192 lesions in each group) in a powered analysis, while our propensity score matching generated 380 lesions (190 lesions in each group), which was almost similar to the estimated sample size. Therefore, the possibility of a Type II error (ß error) might be minimum. Moreover, the standardized difference of PMI between the 2 matched groups was 0.086. Since a standardized difference < 0.1 has been taken to indicate a negligible difference in the prevalence of a covariate between the 2 groups irrespective of the study sample size^36,48^, it is appropriate to mention that use of RA would not increase the risk of PMI in complex lesions.

## Conclusions

In contemporary elective PCI, use of RA was not associated with PMI after a propensity score-matched analysis, while use of RA was associated with greater risk of slow flow. The fact that RA did not increase the risk of myocardial damage in complex lesions would have an impact on revascularization strategy for severely calcified coronary lesions.

## Supplementary Information

Supplementary Tables.

## Data Availability

All data are available from the corresponding author on reasonable request.
