# Supplementary material for: Comparison of the incidence of periprocedural myocardial infarction between percutaneous coronary intervention with versus without rotational atherectomy using propensity score-matching
Source: Sci Rep. 2021 May 27;11:11140. doi: 10.1038/s41598-021-90042-8 (PMC8160267; doi:10.1038/s41598-021-90042-8)
Supplement: Supplementary file 1 — Supplementary Tables. [file 41598_2021_90042_MOESM1_ESM.docx]

**Supplemental Table 1. Comparison of patients, lesions, and procedural characteristics between the RA and the non-RA groups before and after propensity score matching using the match tolerance set as a width of 0.25 multiplied by the SD of the propensity score distribution.**

|  | Before propensity score matching | | | | After propensity score matching | | | |
| --- | --- | --- | --- | --- | --- | --- | --- | --- |
|  |  | | | |  | | | |
|  | RA  group  (n = 203) | Non-RA group  (n = 1147) | P value | Standardized difference | Matched RA  group  (n = 179) | Matched non-RA group  (n = 179) | P value | Standardized  difference |
| Patient characteristics |  |  |  |  |  |  |  |  |
| Age (years) | 74 (62.0-80.0) | 72 (64.0-77.0) | 0.001 | 0.331 | 74.0 (69.0-80.0) | 75.0 (69.0-81.0) | 0.553 | 0.043 |
| Men - n, (%) | 145 (71.4) | 932 (81.3) | 0.002 | 0.235 | 129 (72.1) | 125 (69.8) | 0.727 | 0.051 |
| Overweight (BMI ≥25 kg/m^2^) - n, (%) | 57 (28.1) | 414 (36.1) | 0.031 | 0.172 | 48 (26.8) | 48 (26.8) | 1.000 | 0 |
| Hypertension - n, (%) | 186 (91.6) | 1024 (89.3) | 0.382 | 0.078 | 164 (91.6) | 162 (90.5) | 0.853 | 0.039 |
| Diabetes mellitus - n, (%) | 112 (55.2) | 538 (46.9) | 0.030 | 0.167 | 97 (54.2) | 103 (57.5) | 0.595 | 0.066 |
| Insulin user – n, (%) | 36 (17.7) | 131 (11.4) | 0.015 | 0.179 | 31 (17.3) | 31 (17.3) | 1.000 | 0 |
| Dyslipidemia - n, (%) | 178 (87.7) | 963 (84.0) | 0.206 | 0.106 | 156 (87.2) | 157 (87.7) | 1.000 | 0.015 |
| Current smoker - n, (%) | 20 (10.4)  (n=192) | 200 (18.0)  (n=1111) | 0.009 | 0.219 | 18 (10.7)  (n=169) | 17 (9.6)  (n=177) | 0.859 | 0.036 |
| Chronic renal failure (creatinine >2mg/dl) - n, (%) | 55 (27.1) | 129 (11.2) | <0.001 | 0.413 | 41 (22.9) | 44 (24.6) | 0.804 | 0.040 |
| Estimated GFR (mL/min/1.73m^2^) | 53.5 (11.2-70.0) | 62.9 (47.6-77.8) | <0.001 | 0.433 | 54.6 (31.7-71.2) | 53.8 (22.1-66.9) | 0.498 | 0.072 |
| Chronic renal failure on hemodialysis - n, (%) | 53 (26.1) | 108 (9.4) | <0.001 | 0.448 | 40 (22.3) | 38 (21.2) | 0.898 | 0.027 |
| Statin treatment - n, (%) | 168 (82.8) | 871 (75.9) | 0.037 | 0.171 | 149 (83.2) | 149 (83.2) | 1.000 | 0 |
|  |  |  |  |  |  |  |  |  |
| Lesion characteristics |  |  |  |  |  |  |  |  |
| Reason for PCI |  |  | 0.172 | 0.108 |  |  | 1.000 | 0 |
| PCI to the culprit of ST elevation myocardial infarction - n, (%) | 2 (1.0) | 5(0.4) |  |  | 2 (1.1) | 1 (0.6) |  |  |
| PCI to the culprit of non-ST elevation myocardial infarction - n, (%) | 32 (15.8) | 141 (12.3) |  |  | 27 (15.1) | 28 (15.6) |  |  |
| PCI to the non-AMI lesions - n, (%) | 169 (83.3) | 1001 (87.3) |  |  | 150 (83.8) | 150 (83.8) |  |  |
| Target lesion |  |  | <0.001 | 0.948 |  |  | 0.366 | 0.258 |
| Left main- left anterior descending artery - n, (%) | 131 (64.5) | 502 (43.8) |  |  | 110 (61.5) | 112 (62.6) |  |  |
| Left circumflex artery - n, (%) | 18 (8.9) | 253 (22.1) |  |  | 17 (9.5) | 24 (13.4) |  |  |
| Right coronary artery - n, (%) | 54 (26.6) | 392 (34.2) |  |  | 52 (29.1) | 43 (24.0) |  |  |
| PCI to in-stent restenosis - n, (%) | 15 (7.4) | 125 (10.9) | 0.168 | 0.122 | 14 (7.8) | 16 (8.9) | 0.849 | 0.040 |
| PCI to chronic total occlusion - n, (%) | 4 (2.0) | 120 (10.5) | <0.001 | 0.357 | 4 (2.2) | 5 (2.8) | 1.000 | 0.038 |
| Reference diameter (mm) | 2.39 (2.05-2.71) | 2.37 (2.00-2.83) | 0.853 | 0.012 | 2.39 (2.05-2.72) | 2.33 (2.04-2.76) | 0.582 | 0.073 |
| Lesion length (mm) | 18.78 (11.52-35.02) | 14.84 (10.02-24.62) | <0.001 | 0.301 | 16.55 (10.29-28.57) | 18.49 (11.22-29.46) | 0.924 | 0.023 |
| Lesion angle |  |  | <0.001 | 0.359 |  |  | 0.285 | 0.154 |
| Mild (< 45 º) | 85 (41.9) | 665 (58.0) |  |  | 81 (45.3) | 82 (45.8) |  |  |
| Moderate (45-90 º) | 83 (40.9) | 375 (32.7) |  |  | 68 (38.0) | 77 (43.0) |  |  |
| Severe (> 90 º) | 35 (17.2) | 107 (9.3) |  |  | 30 (16.8) | 20 (11.2) |  |  |
| Calcification |  |  | <0.001 | 1.024 |  |  | 1.000 | 0 |
| None/mild, n (%) | 4 (2.0) | 864 (75.3) |  |  | 4 (2.2) | 4 (2.2) |  |  |
| Moderate/severe, n (%) | 199 (98.0) | 285 (24.8) |  |  | 175 (97.8) | 175 (97.8) |  |  |
| Bifurcation lesion (n=585) |  |  |  |  |  |  |  |  |
| Medina classification |  |  | 0.097 | 0.578 |  |  | 0.809 | 0.897 |
| 1,0,0 | 11 (8.3)  (n=132) | 48 (10.6)  (n=453) |  |  | 10 (9.3)  (n=108) | 11 (9.9)  (n=111) |  |  |
| 1,0,1 | 7 (5.3)  (n=132) | 19 (4.2)  (n=453) |  |  | 6 (5.6)  (n=108) | 3 (2.7)  (n=111) |  |  |
| 1,1,0 | 41 (31.1)  (n=132) | 124 (27.4)  (n=453) |  |  | 32 (29.6)  (n=108) | 34 (30.6)  (n=111) |  |  |
| 1,1,1 | 18 (13.6)  (n=132) | 67 (14.8)  (n=453) |  |  | 14 (13.0)  (n=108) | 15 (13.5)  (n=111) |  |  |
| 0,1,0 | 30 (22.7)  (n=132) | 107 (23.6)  (n=453) |  |  | 25 (23.1)  (n=108) | 23 (20.7)  (n=111) |  |  |
| 0,1,1 | 23 (17.4)  (n=132) | 54 (11.9)  (n=453) |  |  | 19 (17.6)  (n=108) | 19 (17.1)  (n=111) |  |  |
| 0,0,1 | 2 (1.5)  (n=132) | 34 (7.5)  (n=453) |  |  | 2 (1.9)  (n=108) | 6 (5.4)  (n=111) |  |  |
|  |  |  |  |  |  |  |  |  |
| Procedural characteristics |  |  |  |  |  |  |  |  |
| Successful PCI | 203 (100) | 1134 (98.9) | 0.237 | 0.149 | 179 (100) | 174 (97.2) | 0.061 | 0.240 |
| Guiding catheter size |  |  | <0.001 | 0.465 |  |  | <0.001 | 0.240 |
| ≤6Fr - n, (%) | 4 (2.0) | 638 (55.6) |  |  | 4 (2.2) | 28 (15.6) |  |  |
| 7Fr - n, (%) | 180 (88.7) | 427 (37.2) |  |  | 156 (87.2) | 140 (78.2) |  |  |
| 8Fr - n, (%) | 19 (9.4) | 82 (7.1) |  |  | 19 (10.6) | 11 (6.1) |  |  |
| Intra-aortic balloon pump support - n, (%) | 1 (0.5) | 10 (0.9) | 1.000 | 0.048 | 1 (0.8) | 5 (2.8) | 0.215 | 0.151 |
| Use of orbital atherectomy - n, (%) | 0 (0) | 21 (1.8) | 0.060 | 0.191 | 0 (0) | 19 (10.6) | <0.001 | 0.487 |
| Use of scoring balloon or cutting balloon - n, (%) | 73 (36.0) | 301 (26.2) | 0.004 | 0.213 | 69 (47.3) | 77 (43.0) | 0.452 | 0.086 |
| Side branch protection |  |  | <0.001 | 0.259 |  |  | 0.818 | 0 |
| Jailed wire - n, (%) | 81 (39.9) | 262 (22.8) |  |  | 64 (35.8) | 62 (34.6) |  |  |
| Jailed corsair - n, (%) | 7 (3.4) | 32 (2.8) |  |  | 6 (3.4) | 10 (5.6) |  |  |
| Jailed balloon - n, (%) | 2 (1.0) | 8 (0.7) |  |  | 1 (0.6) | 1 (0.6) |  |  |
| Total stent and DCB length | 35.5- (20.0-48.5)  (n=202) | 24.0 (18.0-38.0)  (n=1117) | <0.001 | 0.386 | 32.0 (20.0-44.3)  (n=178) | 30.0 (20.0-45.0)  (n=171) | 0.521 | 0.054 |
| Kissing balloon technique - n, (%) | 0 (0) | 12 (1.0) | 0.232 | 0.142 | 0 (0) | 2 (1.1) | 0.499 | 0.149 |
| Final PCI procedure |  |  | 0.082 | 0.632 |  |  | 0.203 | 0.566 |
| DES- n, (%) | 170 (83.7) | 936 (81.6) |  |  | 147 (82.1) | 144 (80.4) |  |  |
| DCB - n, (%) | 22 (10.8) | 155 (13.5) |  |  | 22 (12.3) | 19 (10.6) |  |  |
| DES + DCB - n, (%) | 8 (3.9) | 18 (1.6) |  |  | 7 (3.9) | 6 (3.4) |  |  |
| BMS - n, (%) | 2 (1.0) | 7 (0.6) |  |  | 2 (1.1) | 1 (0.6) |  |  |
| POBA - n, (%) | 1 (0.5) | 20 (1.7) |  |  | 1 (0.6) | 5 (2.8) |  |  |
| Other - n, (%) | 0 (0) | 11 (1.0) |  |  | 0 (0) | 4 (2.2) |  |  |

Data are presented as a percentage for categorical variables or a median (quartile 1- quartile 3) for nonparametric variables. A Mann-Whitney U test was used for nonparametric continuous variables. A Fisher’s exact test was used for categorical variables.

Abbreviations: RA= rotational atherectomy, BMI = body mass index, GFR = glomerular filtration rate, PCI = percutaneous coronary intervention, AMI = acute myocardial infarction, DCB = drug-coated balloon, TIMI = Thrombolysis in Myocardial Infarction, DES = drug-eluting stent, BMS = bare-metal stent, POBA = plain old balloon angioplasty.

**Supplement Table 2. Comparison of complications between the RA and non-RA groups before and after propensity score matching using the match tolerance set as a width of 0.25 multiplied by the SD of the propensity score distribution.**

|  | Before propensity score matching | | | | After propensity score matching | | | |
| --- | --- | --- | --- | --- | --- | --- | --- | --- |
|  | RA  group  (n = 203) | Non-RA group  (n = 1147) | P value | Standardized difference | Matched RA  group  (n = 179) | Matched non-RA group  (n = 179) | P value | Standardized  difference |
| Periprocedural myocardial infarction – n, (%) | 16 (7.9) | 31 (2.7) | 0.001 | 0.234 | 12 (6.7) | 10 (5.6) | 0.826 | 0.046 |
| Periprocedural myocardial infarction defined by the SCAI criteria – n, (%) | 0 (0) | 2 (0.2) | 1.000 | 0.707 | 0 (0) | 1 (0.6) | 1.000 | 0.110 |
| Periprocedural myocardial infarction defined by the EXCEL criteria– n, (%) | 1 (0.5) | 3 (0.3) | 0.479 | 0.413 | 0 (0) | 2 (1.1) | 0.499 | 0.149 |
| Creatine kinase (U/L) at the next day of PCI | 98  (62.0-156.0) | 80.0  (57.0-120.0) | <0.001 | 0.194 | 96  (62.0-154.0) | 82.0  (57.0-136.0) | 0.169 | 0.080 |
| Slow flow – n, (%) | 34 (16.7) | 101 (8.8) | 0.001 | 0.239 | 29 (16.2) | 13 (7.3) | 0.013 | 0.279 |
| Final TIMI grade of main vessel |  |  | 0.242 | 1.029 |  |  | 0.030 | 1.414 |
| TIMI3 - n, (%) | 202  (99.5) | 1124  (98.0) |  |  | 179 (100) | 173 (96.6) |  |  |
| TIMI≤2 (%) - n, (%) | 1 (0.5) | 23 (2.0) |  |  | 0 (0) | 6 (3.4) |  |  |
| Final TIMI grade of side branch in bifurcation lesions |  |  | 0.303 | 0.300 |  |  | 1.00 | 0.030 |
| TIMI3 - n, (%) | 117 (88.6)  (n=132) | 415 (91.6)  (n=453) |  |  | 97 (89.8)  (n=108) | 100 (90.1)  (n=111) |  |  |
| TIMI≤2 (%) - n, (%) | 15 (11.4)  (n=132) | 38 (8.4)  (n=453) |  |  | 11 (10.2)  (n=108) | 11 (9.9)  (n=111) |  |  |
| Coronary perforation (Ellis type 3) – n, (%) | 0 (0) | 3 (0.3) | 1.000 | 0.926 | 0 (0) | 1 (0.6) | 1.00 | 0.110 |
| Device stuck – n, % | 0 (0) | 0 (0) | - | - | 0 (0) | 0 (0) | - | - |

Data are presented as a percentage for categorical variables or a median (quartile 1- quartile 3) for nonparametric variables. A Mann-Whitney U test was used for nonparametric continuous variables. A Fisher’s exact test was used for categorical variables.

Abbreviations: PCI = percutaneous coronary intervention, TIMI = Thrombolysis in Myocardial Infarction.
